# 440. Detection of COVID-19 Patients Requiring Escalation to ICU Status Using a Naïve Bayes Classifier

**DOI:** 10.1093/ofid/ofab466.639

**Published:** 2021-12-04

**Authors:** William R Barnett, Chad Jaenke, Zachary Holtzapple, James Williams, Nithin Kesireddy, Waleed Khokher, Ragheb Assaly

**Affiliations:** The University of Toledo College of Medicine, Toledo, Ohio

## Abstract

**Background:**

A naïve Bayes classifier is a popular tool used in assigning variables an equal and independent contribution to a binary decision. With respect to COVID-19 severity, the naïve Bayes classifier can consider different variables, such as age, gender, race/ethnicity, comorbidities, and initial laboratory values to determine the probability a patient may need to be admitted or transferred to an intensive care unit (ICU). The aim of this study was to develop a screening tool to detect COVID-19 patients that may require escalation to ICU status.

**Methods:**

Patients hospitalized with COVID-19 were gathered from the end of March 2020 to the end of May 2020 from four hospitals in our metropolitan area. We began searching for potential variables to include in the classification model using chi-square analysis or calculating the optimal cutpoint to separate ICU and non-ICU status. After identifying significant variables, we began using standard procedures to construct a classifier. The dataset was split 7:3 to create samples for training and testing. To appraise the model’s performance, sensitivity, specificity, positive predictive value (PPV), negative predictive value (NPV), area under the curve (AUC), and the Matthew’s correlation coefficient (MCC) were calculated.

Table 1. Univariate analysis of variables in the COVID-19 dataset dichotomized by ICU status

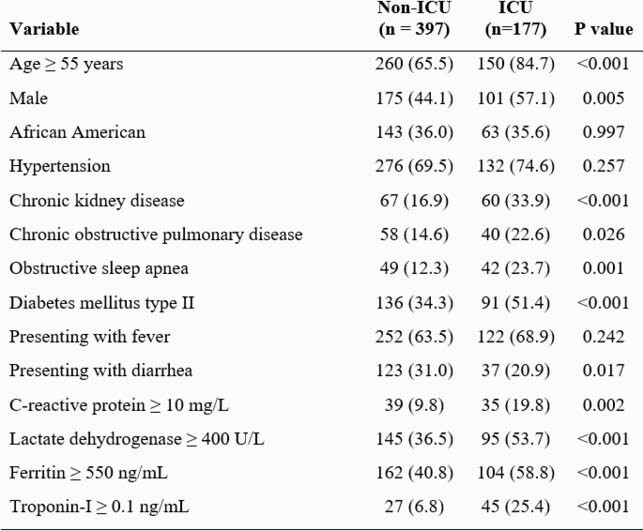

**Results:**

A total of 574 COVID-19 patients were included in the study. There were 402 patients in the training sample and 172 patients in the testing sample. The naïve Bayes classifier demonstrated an overall accuracy result of 75.6% (95% CI; 68.5% – 81.8%) using the 14 variables listed in Table 1. The model was able to correctly classify 84.9% of ICU status patients (sensitivity), but only 54.7% of non-ICU status patients (specificity). The PPV and the NPV were 80.1% and 61.7%, respectively. The AUC was 0.717 (95% CI; 0.629 – 0.805) and the MCC was 0.410.

**Conclusion:**

Our naïve Bayes classifier operates by recognizing certain aspects of severe COVID-19 cases and looking for the probability of the variables in said patients. We present a classification model that potentially could be used alongside other tools to screen patients with COVID-19 early in their hospital course to identify those needing escalation to ICU level care.

**Disclosures:**

**All Authors**: No reported disclosures

